# A comparative evaluation of the role of olfaction in attachment

**DOI:** 10.1007/s10071-024-01891-5

**Published:** 2024-07-30

**Authors:** Emma Cox, Courtney Collins-Pisano, Lane Montgomery, Jeffrey S. Katz

**Affiliations:** Department of Psychological Sciences, Auburn University, 226 Thach Hall, AL, 36849 USA

**Keywords:** Attachment, Odor, Bonding

## Abstract

Olfactory information plays an important role in the attachment and bonding processes for both humans and non-human animals. Odor cues obtained via individual body odor facilitate attachment and bonding processes across species with regard to both mate selection and mother-infant bonding. The purpose of the present paper is to summarize the role of odor as it pertains to bond formation and maintenance in the mother-infant bond for human infants and non-human animal infants, and for mate selection among human adults and non-human animals. We then synthesize this summary with literature on attachment and existing evidence for the relationships between olfaction and attachment processes. Finally, we suggest avenues for areas of future research.

## Introduction

Olfaction plays an important role in the social lives of both humans and non-human animals. All individuals have a unique body odor that reflects the individual’s genetic makeup, overall health, diet, and environmental influences (Brennan and Kendrick 2006; Havlicek and Lenochova 2006; Jacob et al. 2002; Kalmus 1955; Olsson et al. 2014; Wallace 1977; Wedekind et al. 1995; Zuniga et al. 2017). Olfactory signals obtained through body odors influence the development of relationships and bonding processes through mate selection and parent-offspring attachment (Brennan and Kendrick 2006). Regarding mate selection, olfactory information provides cues to individuals’ genetic relatedness, as well as their genetic compatibility (Havlicek and Roberts 2009; Milinski 2006; Weisfeld et al. 2003); in regard to parent-offspring attachment, olfactory cues help guide mother-infant recognition and facilitate bonding (Hepper 1994; Sullivan 2003; Vaglio 2009).

Given the olfactory underpinnings of relationship development and bond formation, it seems reasonable to assume there exists a relationship between olfaction and attachment behaviors. The study of this relationship is emergent and, as such, understanding of the relationships between the two remain limited. The purpose of the present paper is to examine the role of odor in relationship formation and maintenance as it applies to both mother-infant relationships and mate selection in both humans and animals. We then present the existing literature on the relationship between attachment and olfaction and suggest avenues for future research to more fully understand the relationships between these processes.

## Overview of attachment

Relationships play an important role in the lives of both humans and non-human animals. Relationships can take many forms, including familial, friendships, and romantic relationships. These relationships share the common feature of creating attachment bonds between the individuals involved through regular interactions over time (Hinde 1976). As there is currently no uniformly accepted definition of attachment bonds, we here use this term synonymously with attachment to refer to strong emotional ties between individuals (Kobak 2009). From a functional perspective, the development of bonds serves a number of purposes such as providing physical and psychological support, as well as ensuring the survival of newborns (Hazan and Zeifman 1999; Raineki et al. 2010a).

Relationships are often studied from the perspective of attachment theory. Attachment theory was first proposed by John Bowlby in 1969 and suggests that humans are born with a set of behaviors that ensure proximity to attachment figures. In infancy, these behaviors include actions such as crying for attention or movement targeted at reestablishing closeness with the attachment figure (Ainsworth et al. 1978). These behaviors evolve across the lifespan, but always with the goal of establishing proximity to the attachment figure, such that attachment behaviors in adulthood might include talking to an attachment figure or even activating mental representations of an attachment figure (Mikulincer and Shaver 2004; Obegi and Berant 2010). Attachment figures are not inclusive of any person with whom an individual has a relationship, but rather is someone to whom the individual turns to in times of stress and whose presence provides comfort and security to the individual (Bowlby 1969; Obegi and Berant 2010). Attachment figures often act as a secure base for the individual, providing an emotional anchor from which to explore new environments and situations (Feeney & Thrush, 2010).

Attachment has been studied in both non-human animals and humans through examination of the balance between attachment behaviors (e.g., proximity seeking) and exploration behaviors (Ainsworth and Bell 1970). A landmark study by Harlow and Zimmerman (1958) that influenced the development of attachment theory (e.g., Bowlby and Ainsworth) demonstrated secure base use and the importance of maternal comfort in infant rhesus monkeys using an open field test, during which monkeys were placed in an arena with unfamiliar objects and their surrogate mothers. Perhaps the most widely accepted manner of assessing the balance of proximity seeking and exploration behaviors in human infants is through the Strange Situation Procedure. This test was developed by Mary Ainsworth et al. in 1978 and was designed to activate attachment-processes in infants through a series of phases in which an infant is in an unfamiliar room with its mother, with a stranger, and alone. Evaluation of the patterns of behavior displayed by infants during this test highlights individual differences in attachment relationships and has provided support for a classification system of attachment styles based on attachment-exploration balance, as well as infants’ reactions to being reunited with the mother following a period of absence. Ainsworth et al. identified three primary attachment styles: secure (the infant is distressed when the mother is absent, seeks proximity when she returns, and then returns to exploration), insecure-avoidant (the infant is not distressed by the mother’s absence and does not seek proximity when she returns), and insecure-resistant (the infant is distressed by the mother’s absence and engages in excessive proximity-seeking upon her return, but remains inconsolable). A fourth attachment style, insecure disorganized, was later identified by Main and Solomon (1986) to describe children showing a mix of behaviors that do not allow for classification into any of the other attachment styles. This test has also been used to assess attachment behaviors of non-human altricial animals with their human caregivers (cats: Edwards et al. 2007; chimpanzees: Bard and Nadler 1983; dogs: Topal et al. 1998; wolves: Topal et al. 2005), as well as among conspecifics in dogs (Prato-Previde et al. 2009; Riggio et al. 2022). Recently, a new method of evaluating attachment, the Secure Base Test, has become prominent. The Secure Base Test is a modification of Harlow’s open field test and is designed to evaluate secure base use (Masataka 2024; Takeda et al. 2024; Thielke and Udell 2019; Udell et al. 2021; Vitale et al. 2019).

Attachment formation is important for a number of reasons. In humans and altricial non-human animals, attachment formation in infancy is critical to ensure the survival of newborns. Formation of these bonds engenders care from the mother, offers physical protection, and enhances psychological well-being (Harlow and Zimmerman 1958; Raineki et al. 2010a). Attachment formation in infancy has been widely studied in both humans and altricial non-human animals, as patterns of attachment developed during early life persist through adulthood and have important implications for mental health, cognitive functioning, and future social relationships (Bretherton and Waters 1985; Mikulincer et al. 2003; Mikulinver & Shaver, 2012; O’Connor and McCartney 2007). Additionally, attachment processes in both human infants and altricial non-human animals have been found to be highly comparable. For example, evaluation of the mother-infant bond in dogs through the Strange Situation Procedure has demonstrated behavioral patterns similar to human infants with their mothers (Prato-Previde et al. 2009).

Attachment formation in adulthood shares some similarities in function with attachment in infancy. In humans, romantic partners act as attachment figures, providing comfort and security to their partner, as well as social and emotional support (Granqvist et al. 2019; Hazan and Zeifman 1999; McBurney et al. 2006). However, understanding of attachment relationships among adult non-human animals is limited, as few species form lasting relationships with conspecifics (Dolotovskaya, 2020; U.S. National Science Foundation 2013). Prairie voles, a monogamous species, demonstrate preference for their partner over unfamiliar conspecifics, selective aggression towards intruding unfamiliar conspecifics, and coparenting behaviors, suggesting that attachment mechanisms may be activated in monogamous species (Tickerhoof & Smith, 2017).

## Olfaction in bond formation and maintenance

### Mother-infant bond in humans

For human infants, odor learning is of adaptive significance. Human infants are altricial, meaning that they require further development postnatally before they can function independently. This makes the mother-infant bond a critical one. In order to survive, the infant must remain close to the mother and have its needs attended to. Furthermore, this must occur through specific sensory systems, as altricial animals are limited in the sensory inputs they are born with (Moriceau and Sullivan 2005). Resultantly, odor learning begins early within the human lifespan. A predisposition toward odor learning, particularly for the odor of the mother, provides infants with rapid learning of odor associations necessary for survival (Sullivan et al. 2011). In the womb, a fetus receives olfactory information via amniotic fluid that provides information about the mother’s food consumption and scent (Schaal et al. 2020). After birth, infants demonstrate an olfactory preference for amniotic fluid, specifically their own (Schaal et al. 2020). This preference helps guide the infant toward breast feeding via transnatal olfactory continuity, as the odors learned in the womb are also present in the mother’s milk (Schaal et al. 2020). Rapidly, this preference for amniotic fluid diminishes as the odor decreases in relevance. In a study looking at odor preferences in infants, at two days of age there was no difference in preference for the odor of amniotic fluid versus breast milk, but by four days of age newborns preferred the odor of breast milk over that of amniotic fluid (Marlier et al. 2008). Odor continues to be relevant as the infant ages. The transition to solid foods can be guided by the food odors within the mother’s milk (Schaal et al. 2020). Taken together, the results of these studies demonstrate that odor is utilized by the infant in preference formation, specifically concerning food sources.

Beyond the development of odor preferences, odor also plays an important role in emotional regulation of the infant. It has been suggested that odor serves as a hidden regulator of infants’ emotional states (Hofer 2005). Addition or removal of maternal odor can result in up- or down-regulation of emotional processes in the infant, respectively (Hofer 2005). Newborns that were exposed to a familiar odor (i.e., the mother’s milk or vanilla) prior to an aversive experience (e.g., a heel stick) exhibited fewer distress behaviors following the aversive event (Rattaz et al. 2005). Further, infants exposed to the odor of their mother’s milk as the familiar odor showed significantly less agitation during the procedure. In another study by Sullivan and Toubas (1998), both crying and awake (but not crying) infants were presented with hospital gowns saturated with either their mother’s odor, another mother’s odor, or no odor to examine whether the odor of the gown changed infant behavior. Both maternal odors resulted in a reduction in crying among crying infants. However, for both awake and crying infants, only the odor of the infant’s own mother resulted in increases in mouthing (Sullivan and Toubas 1998). This suggests that maternal odor in general can be comforting, but that the odor of one’s own mother has a specific impact on the infant (i.e., induction of feeding behaviors is specific to the infant’s own mother). These studies demonstrate a mediating effect of odor on the behaviors displayed by infants in both aversive and neutral conditions.

In summary, while research on odor learning in infants is limited due to the constraints of working with a vulnerable population, current research indicates that odor learning is prevalent in the early life stages of humans. Within the mother-infant bond, odor is utilized by infants in various ways. Odor guides the formation of food preferences through associations with the mother’s breast milk, creating a safe context from which infants can explore new foods and develop food preferences (Marlier et al. 2008; Schaal et al. 2020). Further, in aversive situations such as blood draws or during crying episodes, both familiar and maternal odors have been shown to decrease the behavioral indicators of distress (Hofer 2005; Rattaz et al. 2005; Sullivan and Toubas 1998). These findings highlight the importance of maternal odor to the developing infant. Maternal odor guides infants to safe foods. It also provides the infant with comfort and security during stressful events, indicative of attachment and bonding processes.

### Mother-infant bond in non-human animals

As with humans, attachment formation is essential to the survival of newborn altricial animals, which rely heavily on their mothers for survival early in life. Newborn altricial animals are predisposed to form attachment to their mothers, as learning to preferentially approach their mother for care improves their probability of survival (Raineki et al. 2010a). Maternal approach is achieved through both imprinting and attachment formation. The process of imprinting involves presentation of a visual stimulus representing a caregiver or even an inanimate object within a short critical window occurring shortly after birth, which triggers a fixed action pattern such as proximity-seeking behavior toward that individual or object (Lorenz 1935). In comparison, attachment formation is a slower process, developed during several longer sensitive periods in which the infant learns to approach a caregiving individual (Robledo et al. 2022). In many species, attachment formation is guided by olfactory learning.

Olfaction is one of the first senses developed in rats, so attachment to mothers is primarily odor-dependent in rat pups (Raineki et al. 2010a; Sullivan 2003). Rat pups utilize their olfactory system to orient toward their mother (Sullivan 2003). Attention of the pups to their mother is promoted by attractant chemosignals present in the mother’s odor (Lin et al. 2005). In rats, attachment formation is supported by a neurological sensitive period during which the olfactory bulb is primed for odor preference formation. Increased norepinephrine release into the olfactory bulb triggers second-messenger systems which promote changes at the glomerular level and encourages memory formation (Coopersmith and Leon 1984; Raineki et al. 2010a). Norepinephrine input comes from the locus coeruleus which is more active and sensitive in rat pups under 10 days old (Raineki et al. 2010a; Sullivan 2003). Odor based attachment has been primarily demonstrated through rat studies, however, evidence in other species exists, including dogs (Hepper 1994), cats (Freeman and Rosenblatt 1978a, b) sheep (Poindron et al. 2007), and goats (Poindron et al. 2007). For example, olfactory cues have also been demonstrated to be sufficient for mother-pup recognition in dogs (Hepper 1994).

Maternal odor information is transmitted both prenatally, through amniotic fluid, and postnatally, through experiences such as pup licking (Raineki et al. 2010). As rat pups have limited mobility at birth, there are limited risks associated with forming attachment to odors in their post-natal environment since they are unlikely to encounter odors besides odors associated with their nest and mother (Raineki et al. 2010a). Resultantly, rat pups display enhanced olfactory activation and preference for odors experienced in the nest (Sullivan et al. 1990). Rat pups can be conditioned to form odor preferences for artificial odors paired with maternal care, suckling, and milk delivery (Brake 1981; Johanson and Teicher 1980). The ability to become conditioned to artificial odors suggests that post-natal learning of maternal odor plays an important role in attachment formation. Further, artificial odors paired with maternal contact and stroking are associated with changes in olfactory bulb activation that support odor preference formation (Wilson et al. 1987). Like human infants, maternal odor plays a role in emotional regulation (Dewaele et al. 2020). Mice exposed to artificial odors perinatally exhibit less anxiety behaviors during an open field task at weaning when tested in the presence of the maternally paired odors (Dewaele et al. 2020).

While young rat pups are predisposed to form odor preferences readily, they do not form odor aversions. During the odor preference sensitive period in the first 10 days following birth, young pups respond to aversive and appetitive stimuli in the same way (Camp and Rudy 1988). Rat pups will approach odors paired with shock in the same manner as they approach odors paired with stroking (Raineki, Moriceau, et al., 2010). This phenomenon likely results from the need to learn to approach maternal odor regardless of the quality of care provided (Bisaz and Sullivan 2012). While rat pups will typically experience occasional poor treatment from the mother such as rough handling, it is important to maintain a strong preference for maternal odor due to the overall benefits of maternal approach behavior, rather than form an aversion due to isolated negative events. Rat pups also learn to approach odors paired with maternal maltreatment (e.g., dragging, throwing, avoiding pups) during the 10-day sensitive period, emphasizing that pups will maintain odor approach behavior to maternal odor despite experiencing poor treatment (Roth and Sullivan 2005). However, despite their predisposition to form attachment, prolonged stress or maltreatment can disrupt rat pups’ maternal attachment and interaction (Moriceau et al. 2009). Cortisol is a byproduct of the HPA axis which is activated by stressful events. Administration of a cortisol injection, utilized to artificially induce a stressful state, is associated with the emergence of odor avoidance to shock paired odors in rat pups even within the odor preference sensitive period in the first 10 days post-birth (Raineki et al. 2010a). The cortisol injection also alters and reduces interactions with the mother by decreasing nursing behavior and increasing amygdala activity (Raineki et al. 2010a). Prolonged experience with an abusive mother is associated with a prolonged increase in rat pup cortisol levels, and results in the same behavioral outcomes and odor avoidance tendencies that are observed with cortisol injections (Raineki et al. 2010a). These findings suggest that olfactory processes may have a role in the type of attachment and strength of the maternal bond formed by rat pups. History of maternal care influences olfactory learning of approach or avoidance for maternal odor, thereby potentially influencing how the rat pup interacts with the mother.

The Major Histocompatibility Complex (MHC) also plays an important role in attachment, as it contributes to the individual odor profile of the mother’s scent. The MHC is considered to be the main genetic controller of individual odor (Brennan and Kendrick 2006). The MHC is a crucial component of the vertebrate immune system. It provides DNA code for antigens that are responsible for recognition of foreign proteins in cells (Havlicek and Roberts 2009). The MHC also determines the proteins excreted to produce individual odor via products such as urine, and may have a role in the sensation and perception of individual odors by influencing which proteins are detected by the olfactory system (Hepper and Cleland 1998). Among rats, moms preferentially retrieve pups with genetically similar MHC over MHC different pups (Yamazaki et al. 2000). For rat pups, cross-fostering alters MHC preference, suggesting that MHC preference is dependent on the nest they are raised in and is not a strictly genetic preference (Yamazaki et al. 2000). It is likely that MHC plays a partial role in attachment formation, but that conditioning to maternal odor is also necessary. Dogs’ ability to recognize their mother but not siblings after extended periods away suggests that there is additional conditioning of maternal odor postnatally and that odor attachment does not solely rely on MHC similarity (Hepper 1994).

In summary, rat and other infant mammal’s predisposition to olfactory learning encourages maternal approach behavior, increasing access to essential survival behaviors such as suckling and grooming. Maternal odor must be paired with quality maternal care to generate approach behavior, whereas extended poor maternal care experience can negatively impact behavior, leading to an aversion to maternal odor. While the role of maternal odor in infant approach behavior has been well studied in rats and some other animals, there is limited understanding of how odor learning influences the strength of attachment bonds formed between infant and animal mothers.

### Mate selection in humans

In adulthood, humans use odor both in relationship formation and in relationship maintenance. Regarding relationship formation, odor plays an important role in attraction both explicitly and implicitly. Explicit use of odor in mate selection involves evaluation of a potential partner’s body odor. Body odor, particularly natural odor (e.g., in the absence of any artificial odor enhancers), has been rated by both men and women as one of the most important characteristics in mate selection (Herz and Inzlicht 2002; Sergeant et al. 2005; White and Cunningham 2017). Additionally, perception of body odor has been shown to influence behavior of both the individual to whom the odor belongs and of smellers of the odor (Roberts and Havlicek 2012). Application of pleasant-smelling odors, such as perfume or deodorant, has been shown to increase self-confidence in both men and women. In a study by Roberts et al. (2009), men were randomly assigned to one of two groups: a deodorant group (who were instructed to wear a provided clinically available deodorant for a four-day period) or a placebo group (who were instructed to wear a placebo deodorant made of alcohol spray with no active ingredients for a four-day period). This study found that men in the placebo group progressively rated themselves as less attractive and self-confident than men in the deodorant group. Additionally, a panel of odor-blind female judges rated videos of the men in the study. Overall, the judges rated the men in the deodorant group as significantly more attractive than the men in the placebo group.

Implicitly, body odor conveys important information regarding a potential mate’s genetic compatibility, biological state, and personality (Gildersleeve et al. 2012; Havlicek and Roberts 2009; Sorokowska et al. 2012). Body odor conveys important information about an individual’s genetic makeup and suitability as a potential mate as a result of the influence of the MHC on individual odor profile.(Brennan and Kendrick 2006) Selection of MHC-dissimilar mates confers important ecological benefits for offspring, by increasing MHC-heterozygosity and thus increasing immune function (Havlicek and Roberts 2009; Penn et al. 2002). Preference for MHC-dissimilarity was first demonstrated in a study by Wedekind et al. (1995) in which women were asked to rate the odor of t-shirts previously worn to sleep by a group of men. The women overwhelmingly rated the odors of men with dissimilar MHC antigens to their own as significantly more pleasant. Interestingly, this effect was mediated by contraceptive use, with women using contraceptive pills preferring the odors of MHC-similar men. Preference for MHC-dissimilarity has since been demonstrated through a number of odor studies in humans (Havlicek and Roberts 2009; Winternitz et al. 2017), though there is evidence that this preference may be mediated by socio-cultural factors (Chaix et al. 2008; Dandine-Roulland et al. 2019).

In addition to genetic compatibility, body odor has been shown to convey information regarding both an individual’s overall health and personality. In regard to health, body odor communicates information pertaining to the quality of an individual’s diet, as well as the presence of illness (diet: Zuniga et al. 2017; illness: Olsson et al. 2014). Additionally, body odor provides biologically relevant cues such as fertility, with men overwhelmingly preferring body odor samples from females on a high fertility day of their menstrual cycle compared to a low fertility day (Gildersleeve et al. 2012). Body odor can also provide some insight into an individual’s personality. In a study by Sorokowska et al. (2012), odor donors ranked themselves on several personality measures. Their odor samples were then ranked by blind observers for these personality measures. There were significant correlations between self-assessed personality traits and odor-based personality judgements, particularly for extraversion, neuroticism, and dominance, suggesting that some personality traits may be detectable by odor alone.

Odor also plays a functional role in the maintenance of relationships. Olfactory ability has been found to positively predict sexual well-being and negatively predict infidelity (Blomkvist et al. 2022). Body odor of a partner can also provide a sense of comfort and security. Both men and women have reported that smelling their partner’s clothing makes them feel happy, comfortable, and secure (McBurney et al. 2006; Shoup et al. 2008). Additionally, presence of a romantic partner’s body odor has been shown to reduce subjective discomfort during stress-inducing events (Granqvist et al. 2019).

Overall, olfactory information gained from the body odor of another individual provides important cues as to that individual’s genetic and biological suitability as a potential partner. This information plays an important role in the initiation and formation of bonds among adult romantic partners. Once these bonds have been established, body odor of a partner provides a sense of comfort and security. The relationship between a partners’ body odor and psychological well-being suggests that there is likely a link between attachment processes and olfaction. Further exploration of this link will provide further insight into the importance of olfactory information in relationship formation and its role in relationship maintenance.

### Mate selection in non-human animals

Investigations into the olfactory bases of mate selection in animals has focused primarily on the MHC. Like humans, animals seeking mates rely on MHC genetic information conveyed in individuals’ odors to enhance genetic diversity of offspring and increase pathogen and parasite resistance (Milinski et al. 2005). The peptides to which MHC proteins bind influences the peptides excreted in urine, which is an important source of odor information for many animal species (Brennan and Kendrick 2006). MHC based mate selectivity has been observed in several animal species including mice, rats, and fish. Female mice make mating choices based on both marking rate and the MHC protein content detected in male urine (Roberts and Gosling 2003). MHC selectivity has been observed in seminatural mice colonies (i.e., outbred mice populations in complex and sizable enclosures which allow for natural social behavior) (Potts et al. 1991), and preference for MHC dissimilar mates has also been observed among laboratory rats (Jordan and Bruford 1998). Female sticklebacks also demonstrate an odor-based preference for water containing MHC peptides that are most optimal for reproduction (Milinski et al. 2005). This preference is reversed after offspring spawn, such that females are more attracted to water with MHC content similar to their own, which may reduce the possibility of raiding nests containing their own offspring (Milinski et al. 2005).

Few animal species participate in long-term relationships akin to human romantic relationships. However, monogamous breeding is common in bird species and requires formation of a bond to maintain long-term commitment to a breeding pair (Emery et al. 2007; Kaplan 2020; Mock and Fujioka 1990). Formation of bonds in long-living avian species may be supported by prosocial behavior that develops in these bird species, similar to prosocial behavior observed in humans (Kaplan 2020). Birds benefit from monogamy by developing familiarity with their mate’s parenting style and reproductive success over successive seasons of mating. Individuals who have bonded with a partner who is a successful parent to their offspring will benefit from establishing a long-term bond, since future mating seasons with that partner will likely also result in additional offspring success (Mock and Fujioka 1990).

These long-term bonds are likely based on more than physical appearance and may involve choices based on factors such as compatibility (Kaplan 2020). Long-term pair bond formation is associated with larger brain size in bird species, suggesting that monogamous relationships require increased cognitive ability (Emery et al. 2007). This increased brain size is especially prevalent in species such as rooks, which have altricial offspring and must cooperate to raise their young (Emery et al. 2007). Rooks also exhibit more active social support among partners, via cooperative actions and coordinated behavior, than monogamous species with smaller brains such as geese (Emery et al. 2007). These findings suggest that long-term bond formation requires greater cognitive demand and is associated with increased effort and quality of the bond.

Thus far, olfactory research on mate selection has focused on genetic contributions to individual odor. The MHC provides information on genetic compatibility which guides an individual’s choice of mate. However, there has been no investigation of the role of odor in relationship formation or maintenance in species which form long-term mating bonds. Further research on the role of olfaction in mate selection of monogamous species is needed to draw more comparisons between human and animal use of odor in bond formation. While both humans and animals utilize the MHC for genetic compatibility information, it is unclear how MHC compatibility or other odor cues might play a role in long-term bond formation and maintenance in non-human animals.

### Linking olfaction and attachment

It is evident that odor plays an important role in the development of attachment bonds across the lifespan for both humans and non-human animals. The relationship between olfactory and attachment processes must be further explored to fully understand the olfactory roots of and implications for attachment processes. Direct explorations of the relationship between these processes are currently very limited, however, some links between attachment and olfaction have been established. Self-reported decreases in olfactory functioning in adult human females has been linked to attachment insecurity (Shell et al. 2021). Additionally, human adults with congenital anosmia report greater social insecurity than healthy controls and women with anosmia report feeling less secure about their partner than healthy controls (Croy et al. 2013).

Presence of an attachment figure’s odor has been shown to confer important psychological benefits. In human infants, presence of the mother’s odor has been shown to reduce discomfort during stressful situations (Rattaz et al. 2005; Sullivan and Toubas 1998). Similarly, human adults report feelings of security and comfort in response to a significant other’s body odor (McBurney et al. 2006; Shoup et al. 2008). Taken together, it seems that body odor of an attachment figure has the potential to provide a secure base effect even in the absence of the individual.

Psychological benefits conferred by olfactory cues may be mediated by the quality of attachment relationships. A study by Granqvist et al. (2019) had human adults rank the discomfort associated with a mild electric shock while exposed to different odors, including the body odor of a romantic partner. Skin conductance and attachment style were also measured. Participants reported less subjective discomfort when their partner’s odor was present. However, skin conductance was only reduced for securely attached individuals. Insecurely attached individuals actually had elevated skin conductance when exposed to their partner’s odor despite reporting feeling a reduction in discomfort. Further supporting the relationship between olfaction and attachment quality, a study by Shell et al. (2022) found that human adults’ disgust ratings of a partner’s body odor differ based on attachment style. Individuals with a dismissive-avoidant attachment style rated their partner’s body odor as equally as disgusting as a stranger’s body odor. Additionally, when compared to individuals with other attachment styles, dismissive-avoidant individuals ranked their partner’s body odor as significantly more disgusting.

These results suggest that the potential secure base effects conferred by an attachment figure’s odor may be beneficial for securely attached individuals only. These findings have important implications. First, for human infants, it has been suggested that the persistence of maternal odor in the absence of the mother serves as a protective factor preventing declines in the mother-infant bond (Hofer 2005). However, if infants’ physiological response to maternal odor is mediated by attachment style, as in adult romantic relationships, this would suggest that, for insecurely attached infants, this persistence of odor may lead to prolonged states of physiological stress. A similar issue may occur with non-human animals. A common practice with companion animals is to provide an article of clothing with the caregiver’s smell in the animal’s crate or designated space to provide comfort. Indeed, dogs have been demonstrated to show more resting behaviors in the presence of the owner’s odor while the owner was physically absent (Horii et al. 2021). However, if this sense of comfort provided by the odor is mediated by attachment style, this practice may not be beneficial to all pets. Further, there is evidence to suggest that affective state of the owner when an odor sample is collected alters dogs’ behavioral responses to their owner’s odor, suggesting that affective state of the owner at the time of wearing the article of clothing to be provided for comfort smelling may have an effect on the item’s ability to provision comfort (D’Aniello et al. 2017). Finally, this practice may not be beneficial for all animal species. Cats, for example, have been shown to demonstrate stress behaviors in the presence of their owner’s odor while the owner is physically absent, highlighting the need for greater understanding of species-specific use of odor in attachment processes (Behnke et el., 2021).

### Future directions

The similarities in function observed in attachment relationships among both humans and altricial non-human animals, as well as the commonality of attachment-based behavioral patterns observed in both newborn humans and altricial non-human animals, suggests that many aspects of the attachment system may be shared across altricial animal species. It is clear that olfactory information plays a critical role in bonding processes across species, however, differences in primary sensory modality may affect the extent to which olfactory information is used. While humans primarily rely on information from visual and auditory inputs, many altricial non-human animals rely on olfaction as their primary sensory modality. For these animals, the relationship between odor and attachment behaviors may be even more pronounced. Further explorations of the relationships between olfaction and attachment will provide important comparative insight into the uses of odor in social processes across species. Additionally, further exploration of the relationships between olfactory cues and attachment related psychological and physiological processes may provide insight into the differential processing of attachment-relevant odors rooted in attachment style.

In human adults, investigations into the relationship between olfaction and attachment have recently begun to emerge. Further evaluation of the mediating role of attachment style on the processing of attachment-relevant odors may provide important insights into differences in cognitive processing and behavioral outcomes associated with these odors. With regard to human infants, it is likely that there exists a similar mediating effect of attachment style on processing of attachment-based odors. Greater understanding of this relationship may allow for early detection and intervention in situations of maladaptive attachment behaviors.

To date there has been no investigation into the link between olfactory functioning and attachment theory in non-human animals. The investigation of the role of olfaction in the formation of different strengths and types of bonds will require the development of methods to test attachment theory for animal-animal bonds both within and across species. The Strange Situation Procedure represents a promising foundation from which to build these methods upon, as it has been previously used to assess animal-animal bonds between dogs living in the same household (Riggio et al. 2022; Sipple et al. 2021) and between mother dogs and their puppies (Prato-Previde et al. 2009). Modification of these methods to include other animal species and to allow for the inclusion of attachment-based odors may reveal functional relationships underlying the cognitive processes between olfaction and attachment.

## Data Availability

No datasets were generated or analysed during the current study.
